# Potential contribution of early endothelial progenitor cell (eEPC)-to-macrophage switching in the development of pulmonary plexogenic lesion

**DOI:** 10.1186/s12931-022-02210-7

**Published:** 2022-10-23

**Authors:** Feng-Jin Shao, Xiao-Ling Guo, Jia-Xue Xu, Rui Liu, Dan-Yue Li, Qing-Hao Li, Ting Zhou, Cun Fang, Xun Tan

**Affiliations:** 1 Department of Veterinary Medicine, Zhejiang University, Hangzhou, 310058 People’s Republic of China; 2 Center for Veterinary Medicine, Zhejiang University, Hangzhou, 310058 People’s Republic of China; 3 Institute of Preventive Veterinary Sciences, Zhejiang University, Hangzhou, 310058 People’s Republic of China; 4 Department of Animal Sciences, Zhejiang University, Hangzhou, 310058 People’s Republic of China; 5 Hainan Institute of Zhejiang University, Sanya, Hainan Province People’s Republic of China

**Keywords:** Pulmonary arterial hypertension, Pulmonary arterial pressure, Plexiform lesion, Nuclear factor erythroid 2-related factor 2, Endothelial progenitor cells, Phenotypic switching, Inflammation, Macrophage, Keap1

## Abstract

**Background:**

Plexiform lesions, which have a dynamic appearance in structure and cellular composition, are the histological hallmark of severe pulmonary arterial hypertension in humans. The pathogenesis of the lesion development remains largely unknown, although it may be related to local inflammation and dysfunction in early progenitor endothelial cells (eEPCs). We tested the hypothesis that eEPCs contribute to the development of plexiform lesions by differentiating into macrophages in the setting of chronic inflammation.

**Methods:**

The eEPC markers CD133 and VEGFR-2, macrophage lineage marker mannose receptor C-type 1 (MRC1), TNFα and nuclear factor erythroid 2-related factor 2 (Nrf2) in plexiform lesions in a broiler model were determined by immunohistochemistry. eEPCs derived from peripheral blood mononuclear cells were exposed to TNFα, and macrophage differentiation and angiogenic capacity of the cells were evaluated by phagocytotic and Matrigel plug assays, respectively. The role of Nrf2 in eEPC-to-macrophage transition as well as in MRC1 expression was also evaluated. Intratracheal installation of TNFα was conducted to determine the effect of local inflammation on the formation of plexiform lesions.

**Results:**

Cells composed of the early lesions have a typical eEPC phenotype whereas those in more mature lesions display molecular and morphological characteristics of macrophages. Increased TNFα production in plexiform lesions was observed with lesion progression. In vitro studies showed that chronic TNFα challenge directed eEPCs to macrophage differentiation accompanied by hyperactivation of Nrf2, a stress-responsive transcription factor. Nrf2 activation (Keap1 knockdown) caused a marked downregulation in CD133 but upregulation in MRC1 mRNA. Dual luciferase reporter assay demonstrated that Nrf2 binds to the promoter of MRC1 to trigger its expression. In good agreement with the in vitro observation, TNFα exposure induced macrophage differentiation of eEPCs in Matrigel plugs, resulting in reduced neovascularization of the plugs. Intratracheal installation of TNFα resulted in a significant increase in plexiform lesion density.

**Conclusions:**

This work provides evidence suggesting that macrophage differentiation of eEPCs resulting from chronic inflammatory stimulation contributes to the development of plexiform lesions. Given the key role of Nrf2 in the phenotypic switching of eEPCs to macrophages, targeting this molecular might be beneficial for intervention of plexiform lesions.

**Supplementary Information:**

The online version contains supplementary material available at 10.1186/s12931-022-02210-7.

## Introduction

Plexiform lesions are a predominant histological feature in humans suffering from severe pulmonary arterial hypertension (PAH), including idiopathic PAH (IPAH), ultimately leading to vascular occlusion [1, 2]. These structures are typically located at an arterial branch point or at the origin of a supernumerary artery, and are considered to be functionally important because it could completely occlude the vessel lumen of the affected vessels [2]. It is believed that patients tend to be unresponsive to vasodilator therapy and have an extremely poor prognosis when plexogenic arteriopathy is present [3].

The cause and origins of plexiform lesions remain largely unclear. Several mechanisms have been proposed, involving inflammation mechanisms and disordered angiogenesis [4–7]. In recent years, endothelial progenitor cells (EPCs) have attracted increasing interest in vascular physiology and pathophysiology [8]. To date, two distinct types of EPCs have been described: the early EPCs (eEPCs, also termed circulating angiogenic cells [ASC] or colony-forming unit-endothelial cells) derived from the bone marrow and the late outgrowth EPCs (late EPCs) derived from nonhematopoietic tissue, presumably from tissue vascular niches [9–11]. A beneficial effect on endothelial repair after injury has, in particular, been shown for early EPCs [12, 13]. Based on more recent observations, however, it is suggested that dysfunction of eEPCs contributes to the formation of plexiform lesions in PAH patients [14–16], which was highlighted by evidence of eEPC accumulation in the sites of lesions. However, the exact mechanisms accounting for eEPC dysfunction and the fate of the EPCs in the sites of plexiform lesions remain to be fully elucidated.

In addition to eEPCs, monocytes/macrophages are also found in increase number within plexiform lesions of human PAH and in animal model with severe PAH [17]. However, it is of interest to note that eEPCs display a mixed macrophage/endothelial cell phenotype [18–20], suggesting complex relationships between eEPCs and monocytes/macrophages. While it is proposed that eEPCs with a mixed macrophage/endothelial cell phenotype can develop an endothelial-like cell phenotype under angiogenic conditions [21, 22], there is also evidence that inflammatory environment induces the differentiation of eEPCs into immune/inflammatory cells by yet unknown mechanisms [23], leading to the suggestion that eEPC angiogenic commitment is not a definitive event and that the phenotype and function of these cells are affected by the local microenvironment. Given the intensive perivascular inflammation of plexiform lesions [7], we proposed that the impaired angiogenic activity of local eEPCs is associated with a phenotypic switching of eEPCs to macrophages. However, evidence supporting this hypothesis is still lacking.

A line of evidence shows that the function and fate of stem and progenitor cells are under redox regulation in physiologic and pathologic conditions [24, 25]. The inducible transcription factor Nrf2 (nuclear factor erythroid 2-related factor 2; encoded by *Nfe2l2* gene) is emerging as a central regulator of oxidative stress by activating a wide array of cytoprotective and antioxidant gene targets [26–29]. Although initially considered to function primarily for maintaining and regulating the cellular redox equilibrium, Nrf2 is now recognized to modulate various cellular processes including cell proliferation and differentiation [30]. Recent evidence shows that Nrf2 contributes to the pathogenesis of atherosclerosis via inducing the phenotypic changes of vascular cells as well as macrophages in the lesion [31, 32]. In this regard, Nrf2 may serve as a signaling mechanism to participate in the pathogenesis of plexiform lesions.

Although numerous experimental animal models of PAH have been developed, only few of them develop plexiform-like lesions (reviewed by Bonnet et al. 2017) [33]. In addition, attempts to better understand the pathogenesis of plexogenic arteriopathy in humans with severe PAH have been hampered by the absence of animal models in which plexiform lesions develop spontaneously [34]. Domesticated fast growing meat-type chickens (broiler chickens) are highly prone to idiopathic PAH (previously known as ascites syndrome; pulmonary hypertension syndrome) [35–37], and can spontaneously develop plexiform lesions in small pulmonary arteries exhibiting histological features similar to that seen in human PAH [38–40]. We have recently confirmed the presence of eEPCs (CD133+/VEGFR-2+ cells) and foam-like macrophages in the structures, and provided evidence that the lesion development in this avian model is associated with hemodynamic stress [41, 42]. More recently, we showed that transplantation of mesenchymal stromal cells attenuates neointimal and plexogenic arteriopathy in PAH broiler chickens through modulating lung inflammation [43].

The aim of this work is to uncover the mechanisms by which inflammation and eEPCs conspire to cause the plexiform lesions in the avian model. We demonstrate here that chronic inflammation leads to the phenotypic switching of chicken eEPCs into macrophage lineage resulting in reduced angiogenic potential that involves the transcription factor Nrf2. Moreover, intratracheal instillation of proinflammatory cytokine tumor necrosis factor α (TNFα) promotes the development of plexiform lesions in broilers. This provides new insight into the mechanisms by which inflammation and eEPCs contribute to the plexogenic arteriopathy.

## Materials and methods

### Animal ethics

The animal experiments followed the National Guidelines for the Ethical Review of Laboratory Animal Welfare and were approved by the Ethics Committee of the Zhejiang University (ZJU20170554).

### Animals

Cobb-500 broiler chickens with mixed sex were obtained at 1-day old from a local commercial hatchery (Hangzhou, China) and were reared at thermoneutral temperatures. They were fed a 21% crude protein corn-soybean meal-based broiler ration formulated to meet or exceed the NRC (1994) standards for all ingredients. Feed and water were supplied ad libitum.

### Histology and immunohistochemistry

Birds at 4 weeks of age were humanly killed by cervical dislocation. The whole right lung was collected and cut in the transverse plane at the major rib indentations (costal sulci). For histological study, one inter-rib division from the middle of each lung was fixed in 4% paraformaldehyde. The apical regions of the left lungs were stored in liquid nitrogen until use. The paraffin-embedded blocks were serially cut in the transverse plane at 4–5 μm thickness. One slide of each lung was stained with haematoxylin and eosin (H&E). Number of plexiform lesions was counted for calculation of lesion density (number of lesions per section/cm^2^ per section).

The procedure for immunohistochemistry has been previously described [42]. Lung sections were incubated with mouse anti-chicken CD133 (self-prepared), rabbit anti-rat VEGFR-2 (Boster Biotechnology Technology, China), mouse anti-chicken monocyte/macrophage MRC1 (KUL-01, Southern Biotech, Birmingham, USA), rabbit anti-human Nrf2 (Proteintech, Wuhan, China), or mouse anti-human TNFα (Huabio, Hangzhou, China) at a dilution of 1:50–1:200. The primary antibody detection was performed by using appropriate horseradish peroxidase (HRP)-conjugated secondary antibody and visualized with DAB (3,3′-diaminobenzidine tetrahydrochloride), followed by counterstaining with haematoxylin.

### Ex vivo expansion of eEPCs

Ex vivo expansion of eEPCs were performed exactly as described in our previous work [44]. Briefly, mononuclear cell fraction of the peripheral blood (PBMC) from 4-week-old healthy birds was cultured in Endothelial cell growth medium (EGM)-2 (Lonza, Walkersvil, MD, USA) containing 2% fetal bovine serum (FBS), 100 U/ml penicillin, and 100 μg/ml streptomycin at 39 °C in 5% CO_2_. Non-adherent cells were removed after 48 h. On day 4 of culture, cells were passaged using 0.25% trypsin/EDTA (Invitrogen) and plated on rat tail type 1 collagen-coated 6-well plates at 1 × 10^7^ cells/well.

### Cell viability assay

Cell viability was measured by using Cell Counting Kit-8 (CCK-8, FDbio Science, Hangzhou, China) according to the manufacturer' instructions. Briefly, eEPCs were seeded in 96-well plates at 1 × 10^4^ cells/well in the presence or absence of r-TNFα (PeproTech Inc., Rocky Hill, NJ, USA). At the indicated time points, 10 μl of CCK8 solution was added into each well, followed by incubation of the plates at 39 °C in 5% CO_2_ for 2 h. The optical density (OD) was measured at 450 nm.

### Dil-ac-LDL and lectin staining

eEPCs were characterized by their ability to take up 1,1-dioctadecyl-3,3,3,3-tetramethylindocarbocyanine-labelled acetylated low-density lipoprotein (Dil-Ac-LDL) and bind to lectin (Ulex europaeus agglutinin, Sigma-Aldrich, Shanghai, China). A detailed protocol for Dil-Ac-LDL/lectin labeling has been described previously [45]. Cells were observed with a fluorescence microscope and photographed. The double-labelled cells were identified as eEPCs [46].

### Immunocytochemistry (ICC)

Cells were fixed with 4% paraformaldehyde, followed by antigen retrieval (for MRC1) in Tris–EDTA buffer (pH 9.0) or permeabilization (for Nrf2 and CD133) in 0.1% Triton X-100 for 5–10 min. Nonspecific binding sites were blocked with 10% fetal bovine serum (FBS) for 20 min, followed by incubation with an anti-MRC1 (Southern Biotech, Birmingham, USA) used at a dilution of 1:50, anti-chicken CD133 (self-prepared) or anti-Nrf2 (Proteintech, Wuhan, China) at 1:200 overnight at 4 °C. The primary antibody detection was performed with an FITC-labeled or Alexa Fluor 568-conjugated secondary antibody (Abcam, Cambridge, UK). Nuclei were visualized with DAPI (4′-6-diamidino-2-phenylindole) for 5 min. Cells were observed with a fluorescence microscope and photographed. MRC1+ and CD133+ cells in each well were counted in 6 randomly selected high-power fields (× 200).

### Phagocytosis assay

eEPCs were cultured in 24-well plates at 3 × 10^5^ cells/well with or without recombinant TNFα (r-TNFα; 50 ng/ml) for 3–6 days. Cells were then marked with DAPI for 3 h before incubation with FITC-labeled *E. coli* DH5α (1 × 10^9^ CFU/ml) for another 3 h. Extracellular bacteria was killed by gentamicin. Cells that phagocytosed bacteria were counted in at least 6 randomly selected high-power fields (× 200) under a fluorescence microscope. Broiler PBMNC-derived macrophages were used as a positive control.

### In vivo Matrigel plug assay

Briefly, 6 × 10^5^ eEPCs were treated with or without recombinant TNFα (r-TNFα; 50 ng/ml) for 24 h and were mixed with 200 μl Matrigel (BD Biosciences, San Jose, CA, USA). Thereafter, the Matrigel mixture was subcutaneously injected into 4-week-old broilers. Matrigel mixed only with r-TNFα was used as a blank control. After 6 days, the birds were killed and the Matrigel plugs were harvested, fixed in 4% formaldehyde and processed for histology and immunohistochemistry analyses.

### Keap1 siRNA transfection

Chicken nontargeting negative control siRNAs (NC siRNA) and siRNA specific for the chicken Keap1 was designed and synthesized by GenePharma (Shanghai, China). Briefly, siRNA was complexed with Lipofectamine™ 2000 (Invitrogen, Waltham, MA, USA) according to manufacturer's instructions before transfection. eEPCs were incubated with siRNA (final siRNA pool concentration of 20 nM) for 6 h in a humidified incubator. Knockdown efficiencies were determined by qPCR.

### Real-time quantitative PCR assay

Total RNA was extracted from the cultured cells using TRIzol (Takara). The RNA was reverse transcribed with the PrimeScript RT reagent Kit with genomic DNA (gDNA) Eraser (Takara, Dalian, China). The 80–150-bp primers for each gene were purchased from Tsingke Biological technology (Additional file 1: Table S1). Quantitative assessment of target messenger RNA (mRNA) levels was performed by qPCR using a SYBR-Green Quantitative PCR kit (Vazyme, Nanjing, China) with a Roche LightCycler 480 II system (Roche Diagnostics GmbH, Mannheim, German). The cycle threshold (Ct) values were normalized to the expression of two reference genes (*B2M*, *RPL19*). The relative expression of mRNA was calculated using a Pfaffl analysis method.

### Protein extraction and Western blot analysis

Total protein extraction from lung tissues and cultured cells were performed by using a radioimmunoprecipitation assay buffer (RIPA) containing protease inhibitors and phosphatase inhibitors (FDbio Science, Hangzhou, China). Nuclear and cytoplasmic fractionation was conducted using a Nuclear-Cytosol Extraction Kit (FDbio Science, Hangzhou, China) according to the manufacturer’s instructions. Samples were boiled at 99 °C for 5 min and then separated by using a sodium dodecyl sulfate (SDS)-10% polyacrylamide gel electrophoresis (PAGE) Fast Preparation Kit (FDbio Science, Hangzhou, China). ​After gel electrophoresis, the proteins from the gels were transferred to the 0.45 μm PVDF membranes (Millipore, USA). The membranes were blocked in 5% non-fat milk for 2 h at room temperature and incubated with primary antibodies against Nrf2 (Proteintech, Wuhan, China), Keap1 (Proteintech, Wuhan, China), β-actin (Santa Cruz Biotechnology, Shanghai, China), histone (Santa Cruz Biotechnology, Shanghai, China) and tubulin (FDbio Science, Hangzhou, China) at a dilution of 1:1000 overnight at 4 °C. The primary antibody was detected by a horseradish peroxidase (HRP)-conjugated secondary antibody (FDbio Science, Hangzhou, China). Electrochemiluminescent (ECL, FDbio Science, Hangzhou, China) was used to visualize the immunoreactive bands.

### Luciferase assay

The full-length open reading frame (ORF) of chicken Nrf2 cDNA with an optimal Kozak consensus sequence just before the in-frame first ATG was cloned into the eukaryotic expression vector pEGFP-C3 (pEGFP-C3-Nrf2). A predicted regulatory region containing 978 kb of a 5′ flanking sequence (from − 45 to − 1022) of *MRC1* was cloned into the K*pn* I and H*ind* III of pGL3-basic vector (Promega, Madison, Wisconsin, USA) containing a firefly luciferase reporter gene. The constructs were confirmed by DNA sequencing. The pRL-TK vector (Promega, Madison, Wisconsin, USA) containing a Renilla reniformis luciferase reporter gene was used as a control for transfection efficiency in the Dual-Luciferase Reporter Assay System. All plasmid DNAs were transfected into the HEK-293T cell line using the Lipo8000 (Beyotime Biotech, Nanjing, China). Luciferase activities were analyzed in 20 μl cell lysates with the Dual Luciferase Assay kit (Vazyme, Nanjing, China) on a BioTek Synergy H1 microplate reader (Winooski, Vermont, USA). The relative luciferase activities are expressed as a ratio of the pGL3 reporter activity to that of the control plasmid pRL.

### Intratracheal instillation of TNFα

Intratracheal TNFα instillation was performed following a modified protocol described earlier for rat [47]. In brief, birds at 14 days of age were anesthetized by inhalation of diethyl ether and received twice either 100 μl saline or 0.5 μg recombinant TNFα in 100 μl saline solution intratracheally, followed by another intratracheal instillation after 3 days. Birds were humanly killed 3 days after the second instillation. The hearts were removed, dissected, and weighed for calculation of the right-to-total ventricular weight ratio (RV/TV) as an indicator of pulmonary arterial hypertension [37]. Lung tissue was sampled as described above.

### Statistical analysis

Data were analyzed for normality using Shapiro–Wilk test as a justification for using parametric analysis. Difference in cell proliferation was compared using one-way analysis of variance (ANOVA) followed by Bonferroni post-hoc test. Other data were analyzed using non-parametric Mann–Whitney U test due to the small sample size or not normally distributed. Data were expressed as mean ± s.d. (ANOVA) or median ± 95% confidence interval (Mann–Whitney). The software used was SPSS 22.0 for Window (IBM Corp., Armonk, NY, USA). Differences were considered significant at *P* < 0.05.

## Results

### Cells within the early and mature plexiform lesions demonstrate distinct molecular characteristics

**Fig. 1:**
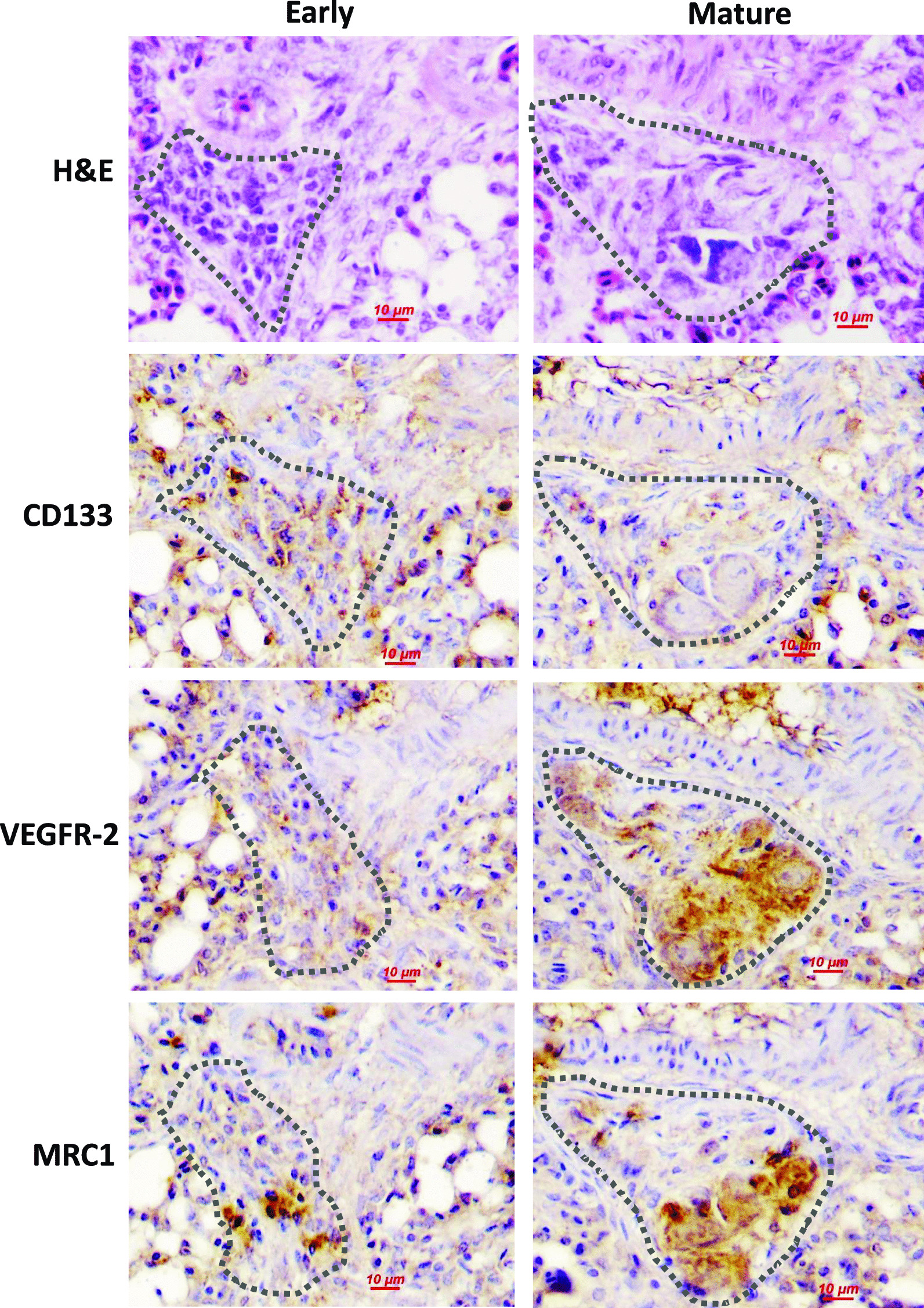
Characterization of the phenotypes of cells within plexiform lesions. Representative serial hematoxylin and eosin (H&E)-stained sections and immunohistochemistry images showing histologic features and immunostaining results of plexiform lesions at different maturing stages from 4-week-old broiler chickens. The cells in the early immature lesion (left) exhibit endothelial-like morphology and those in more mature lesion (right) consist predominantly of foam-like macrophage cells. Immunohistochemistry analysis were performed using primary antibodies against monocyte/macrophage marker mannose receptor C-type 1 (MRC1), stem/progenitor cell marker CD133 and vascular endothelial growth factor receptor (VEGFR)-2. All immunostained sections are counterstained with hematoxylin. The dotted lines represent the edge of lesions

To evaluate the cell phenotype in plexiform lesions at different differentiation stages, lung slides was examined to confirm the presence of plexiform lesions by H&E stain, and the expression of CD133, VEGFR-2 as well as mannose receptor C-type 1 (MRC1), was evaluated by immunohistochemistry analysis using serially cut slides. MRC1+ monocyte/macrophage lineage cells have been found to exhibit features similar to those in mammals [48]. As shown in Fig. 1, we determined strong expression of MRC1, CD133 and VEGFR-2 in cells within the early lesions indicative of the presence of a mixed macrophage/endothelial cell phenotype that has been defined as eEPCs. In more mature lesions foam-like macrophages became predominant, which demonstrated weak CD133 reactivity but strong expression in MRC1 and VEGFR-2, resembling differentiated, polarized macrophages in mammals [49].

### TNFα production in plexiform lesions increases with lesion progression

**Fig. 2:**
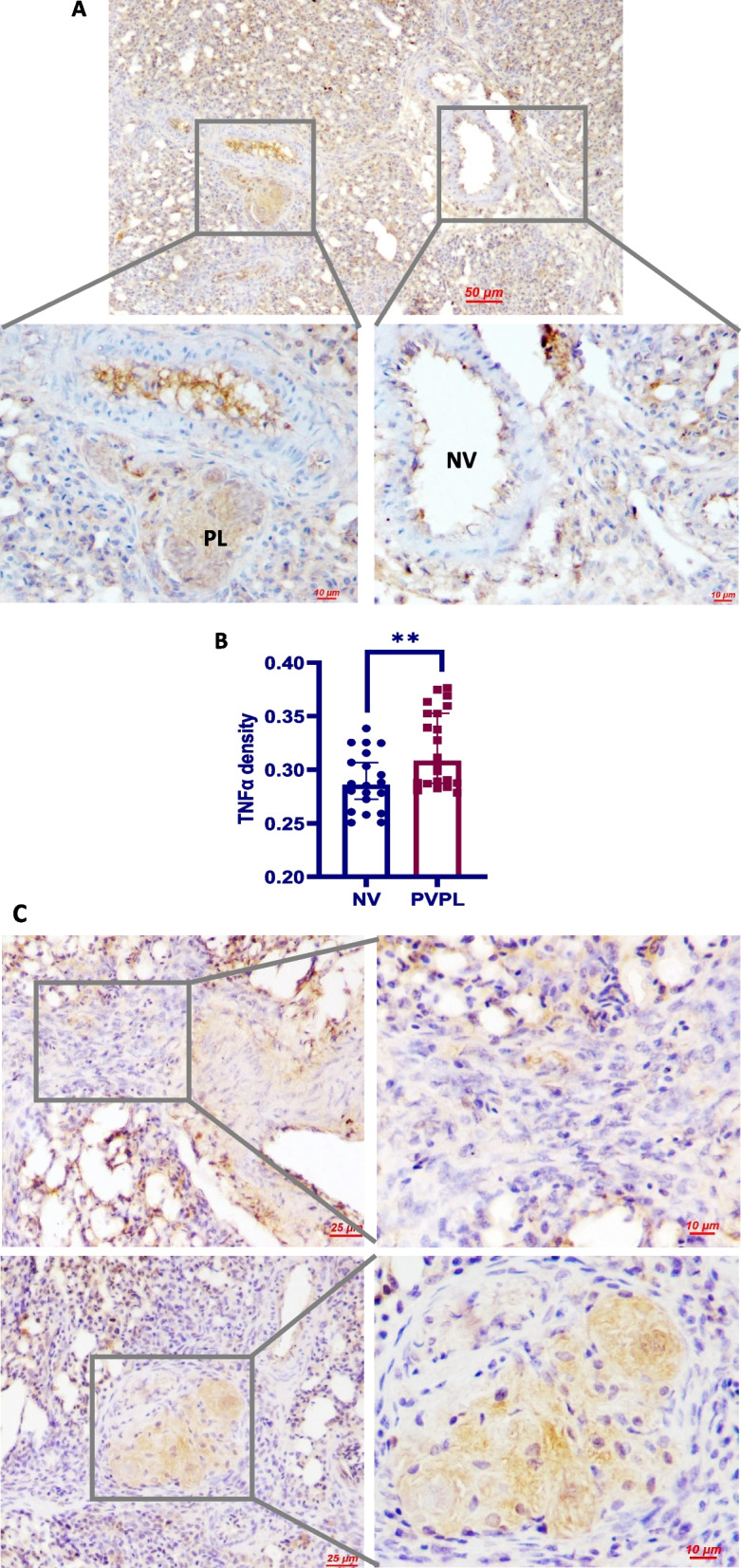
Immunohistochemistry analysis of TNFα. **A** Normally branching vessels showed weak intimal expression of TNFα (right panel) while the arteries from which plexiform lesions arose displayed stronger endothelial signal of TNFα (left panel). *NV* normally branching vessels. *PL* plexiform lesion. **B** Semi-quantification analysis of TNFα in normally branching vessels (NV) and parent vessels of plexiform lesions (PVPL) by measuring the optical density. Data are expressed as median ± 95% confidence interval of at least 20 arterioles. **P < 0.01. **C** The EPCs in early lesions (up) showed limited expression of TNFα as compared to the foam-like macrophage in mature lesions (down)

To confirm that the development of plexiform lesions in our avian model is associated with aberrant TNFα production, an immunohistochemical study of histological sections was performed. As expected, the parent vessels of plexiform lesions had increased signal of TNFα in the endothelial cell layer than the vessels without lesions (Fig. 2A, B). In addition, we noted a progressive increase in stromal TNFα production with the lesion progression (Fig. 2C).

### Chronic TNFα exposure promotes the differentiation of eEPCs to macrophages

**Fig. 3:**
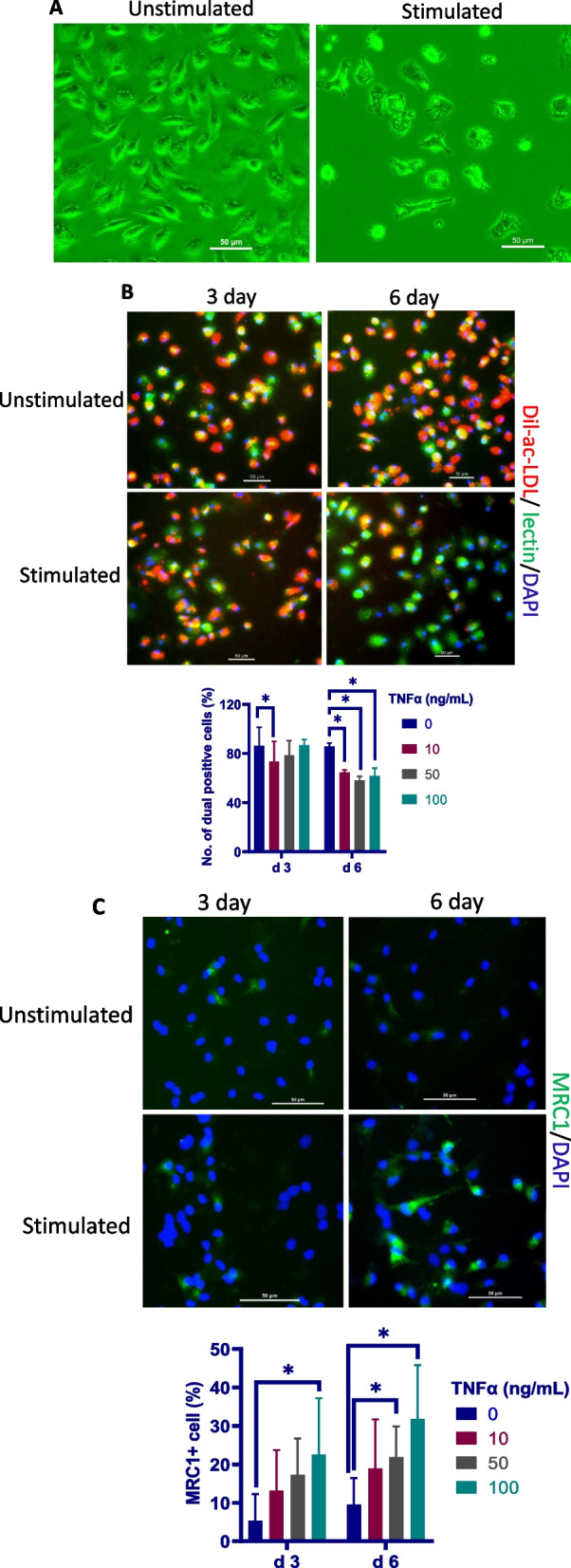

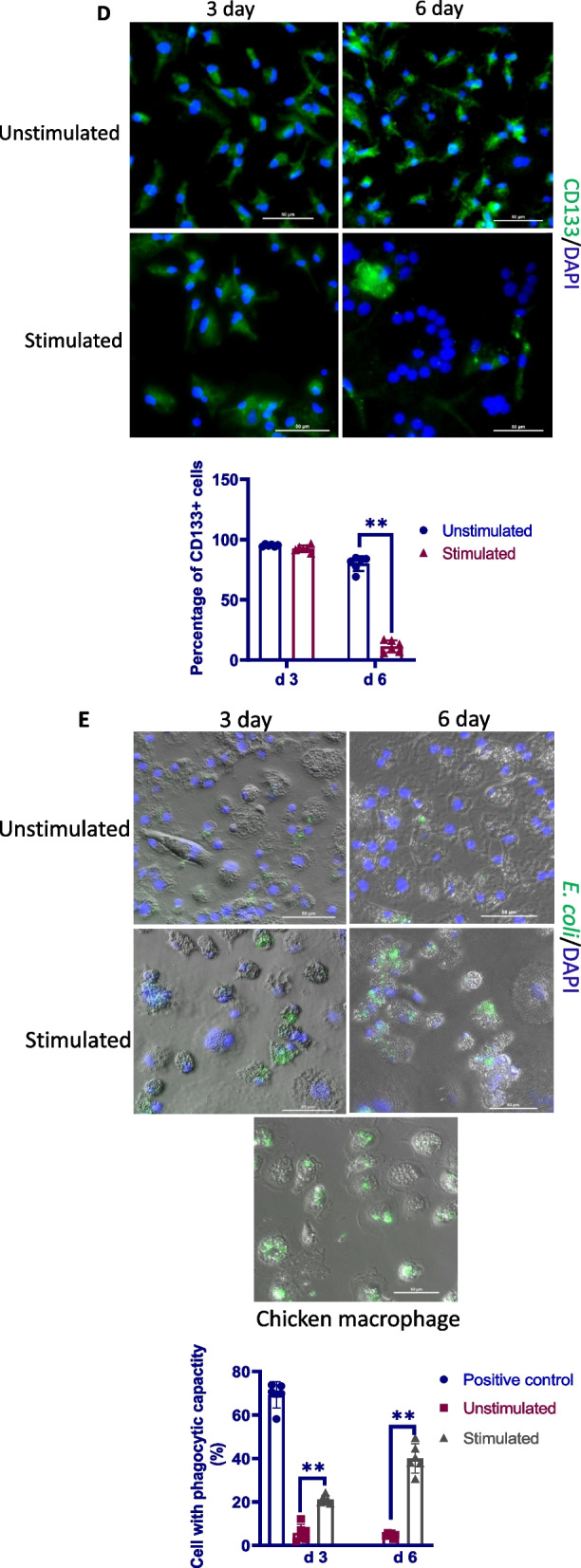
Chronic exposure to TNFα leads to eEPC-to- macrophage conversion. **A** Representative photographs showing the morphological change of eEPCs in response to chronic TNFα exposure. Cells were incubated with TNFα at 50 ng/ml for 6 days. **B** Characterization of eEPCs. eEPCs were subjected to fluorescence staining with Dil-ac-LDL (red) and lectin (green). Cell nuclei were visualized by DAPI (blue). Dual positives were counted (*n* = 3). **C** Characterization of macrophage lineage. eEPCs were subjected to fluorescence staining with anti-KUL-01 (green). The cell nuclei were labeled with DAPI (blue). MRC1+ cells were counted (*n* = 6). **D** eEPCs were exposed to TNFα at 50 ng/ml for 3–6 days and subjected to immunofluorescence analysis with anti-CD133 (green). The cell nuclei were labeled with DAPI (blue). CD133+ cells were counted (*n* = 6). **E** Phagocytosis capability assay. eEPCs were exposed to TNFα at 50 ng/ml for 3–6 days and allowed to phagocyte *E. coli* (green). The cell nuclei were labeled with DAPI (blue). Chicken PBMNC-derived macrophages were used as a positive control. Cells having intracellular bacteria were counted (*n* = 6). The data are representative of 2 separate experiments. Photographs are from one representative experiment. Data were expressed as median ± 95% confidence interval (Mann–Whitney). *P < 0.05. **P < 0.01

Although TNFα has been shown to induce cell death in many cell types [50], we did not determine a significant effect of TNFα at 10–100 ng/ml on cell viability (Additional file 2: Fig. S1). Nevertheless, a remarkable morphologic change was observed in TNFα-challenged eEPCs, characterized by loss of the typical spindle-shaped eEPC appearance and acquisition of rounded and loosely attached phenotype showing numerous superficial dendrites (Fig. 3A), matching the morphology of mature macrophages. In line with the morphologic change, the portion of cells with an eEPC phenotype, as defined as acLDL+/lectin+ cells [18, 51], was markedly declined following exposure to TNFα for 6 days (Fig. 3B). In contrast, MRC1 + cells were significantly increased while CD133+ cells were decreased in the same cultures (Fig. 3C, D). To further confirm that chronic inflammation drives the differentiation of eEPCs to macrophage lineage, phagocytosis assay was performed. As shown in Fig. 3E, ePECs acquired strong phagocytic capability in uptake of FITC-labeled bacteria following treatment with TNFα for 6 days. Together, the data suggest that chronic TNFα challenge triggers the differentiation of eEPCs to macrophages.

### Chronic exposure to TNFα attenuates in vivo angiogenic potential of eEPCs

**Fig. 4:**
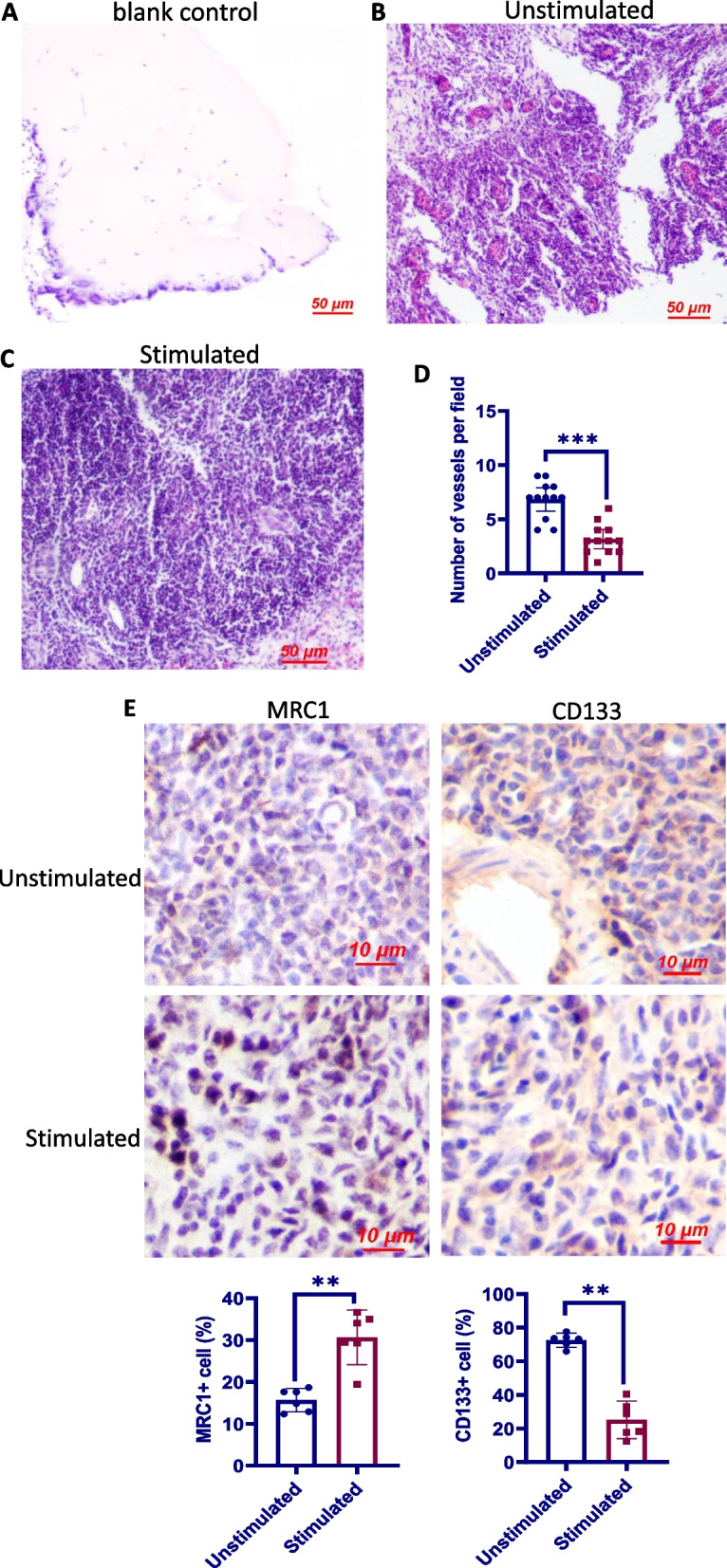
In vivo angiogenic potential and phenotype of eEPCs exposed to TNFα. **A–C** Representative photographs showing the morphology of subcutaneous Matrigel plug containing TNFα (blank control), eEPCs (control) and both (TNFα). Injection of eEPCs without TNFα led to the formation of capillary-like structures containing many erythrocytes. **D** Quantification of the newly-formed vessels in the Matrigel containing eEPCs with or without TNFα. Vessel density was expressed as the number of vessels per field (× 400) (*n* = 6). **E** Immunohistochemical staining of paraffin-embedded Matrigel plug was performed by using the anti-KUL-01 and anti-CD133 antibody. Positive cells were automatically identified and counted in 6 randomly selected fields (× 400) by using ImageJ (version 1.52). Bars are median ± 95% confidence interval. The data are representative of 2 separate experiments

We next evaluated the effects of chronic TNFα exposure on the angiogenic potential of eEPCs by using an in vivo Matrigel plug assay. H&E staining revealed the presence of numerous lumenal structures containing erythrocytes in the plugs with eEPCs; however, in the presence of TNFα, the number of vascular structures in the plugs containing eEPCs was significantly reduced (Fig. 4B–D). Of note, few host cells invaded the plugs containing Matrigel only or Matrigel with r-TNFα, although giant cells were found to infiltrate into the borders of the implants (Fig. 4A), indicating that eEPCs within the implants were not contaminated with host cells (Fig. 4B, C). Plug sections were also subjected to immunohistochemistry analyses of the expression of MRC1 and CD133. Similar to the in vitro results, the Matrigel plugs containing TNFα and eEPCs had increased MRC1+ but decreased CD133+ cells than those containing eEPCs alone (Fig. 4E).

### Nrf2 is essential for the phenotypic changes of eEPCs to macrophages in response to chronic TNFα stimulation

**Fig. 5:**
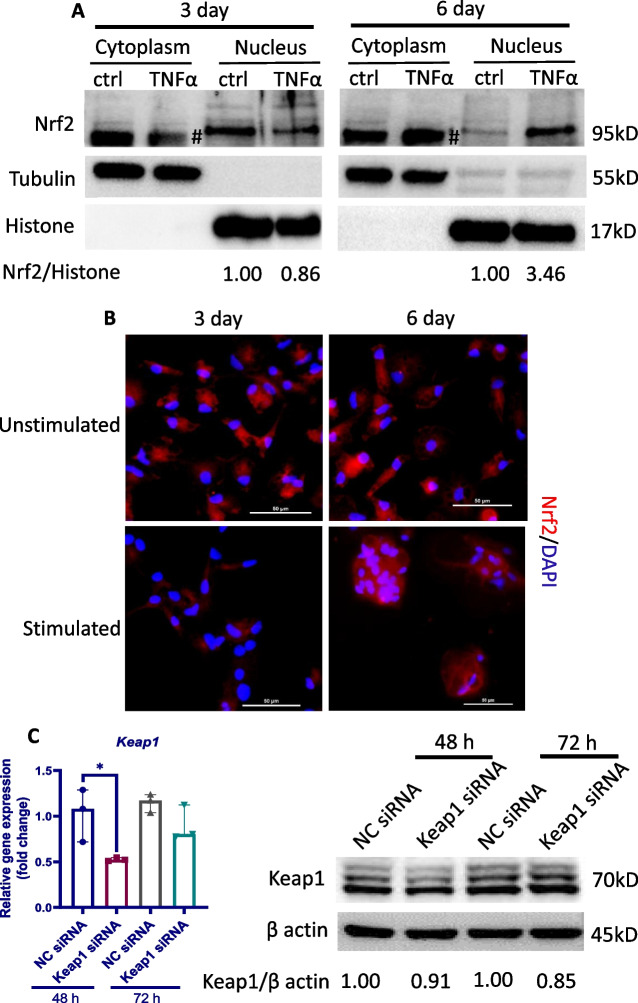

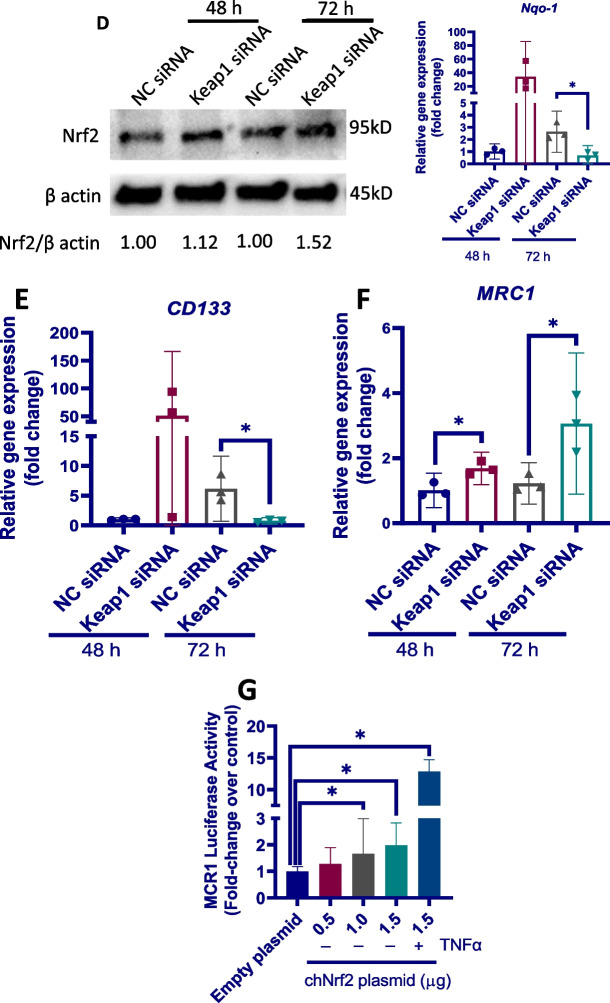
Nrf2 regulates the eEPCs-to-macrophage conversion in response to chronic TNFα stimulation. **A** eEPCs were exposed to 50 ng/ml TNFα for the indicated time. Total cytoplasmic and nuclear proteins were extracted for Western blot using an anti-Nrf2. Tubulin and Histone are shown as loading control, respectively. # non-specific bands. **B** Alternatively, cells were subjected to immunofluorescence analysis of Nrf2 nuclear translocation. Nrf2 was probed with a primary anti-Nrf2 antibody and visualized with an Alexa Fluor 594-conjugated secondary antibody (red). Cell nuclei were stained with DAPI (blue). **C–F** eEPCs were transfected with a siRNA targeting chicken Keap1 or a negative control (NC) siRNA for 48–72 h. **C** Evaluation of knockdown efficiency of Keap1. Total RNAs and whole-cell lysates were subjected to qPCR and Western blot analysis, respectively, for determination of Keap1 mRNA (left panel, *n* = 3) and protein levels (right panel). **D** Evaluation of Nrf2 activation after siRNA silencing of Keap1. Total RNAs and whole-cell lysates were subjected to Western blot and qPCR analysis, respectively, for determination of Nrf2 protein levels (left panel) and relative mRNA level of Nrf2 target genes Nqo-1 (NAD(P)H:quinone oxidoreductase 1) (right panel, *n* = 3). **E–F** Effect of Nrf2 activation on the mRNA expression of stem/progenitor cell marker CD133 and macrophage marker MRC1 (*n* = 3). **G** HEK-293T cells were co-transfected with the chNrf2 expression vector pEGFP-C3-Nrf2 (denoted in μg of DNA per well of a 12-well plate) and the plasmid constructs containing a firefly luciferase gene under the control of the MRC1 promoter along with the pRL-TK Renilla. Empty pEGFP-C3 was used as control. Cells were incubated with or without TNFα (50 ng/ml) for 72 h and processed for measurement of luciferase activity (*n* = 3). The data are representative of at least 2 separate experiments with similar results. Data were expressed as median ± 95% confidence interval. *P < 0.05

Nrf2 has been previously reported to regulate macrophage phenotype in response to oxidative stress and chronic inflammation [31]. On activation, Nrf2 translocates into the nucleus to exert a variety of biological functions [52]. To determine whether Nrf2 regulates phenotypic switching of eEPCs in response to chronic inflammation, we first determined by Western blot the protein level of Nrf2 in the cytoplasmic and nuclear fractions prepared from eEPCs after treatment with TNFα (50 ng/ml) for 3–6 days. As shown in Fig. 5A, Nrf2 protein was detected only in the nuclear fractions from both normal and TNFα-exposed cells. Interestingly, nuclear Nrf2 level tended to be decreased after treatment with TNFα for 3 days, followed by a marked increase at day 6. In line with the Western blot analysis, immunofluorescent staining demonstrated strongly increased nuclear Nrf2 signal in cells exposed to TNFα for 6 days (Fig. 5B).

To confirm Nrf2 overactivation drives phenotypic switching of eEPCs, we modulated Nrf2 activity in eEPCs by genetic knockdown of *Keap1*, which targets Nrf2 for ubiquitination and subsequent degradation in cytoplasm [53]. siRNA silencing of Keap1 (Fig. 5C) resulted in a persistent activation of Nrf2, as evidenced by increased Nrf2 accumulation in the whole cell lysates and upregulated expression of its target gene *Nqo-1* (NAD(P)H:quinone oxidoreductase 1) (Fig. 5D). The effects of *Keap1* knockdown on *CD133* and *MRC1* expression were also evaluated. We noticed a marked upregulation in CD133 mRNA level during the first 48 h following Nrf2 activation; however, an opposite effect was observed when Nrf2 activation was sustained for 72 h (Fig. 5E). Interestingly, *Keap1* knockdown resulted in a persistent upregulation in *MRC1* (Fig. 5F). Based on this finding, we hypothesized that Nrf2 regulates the promoter of *MRC1*. Results from dual luciferase reporter assay showed that increasing chNrf2 plasmid upregulated the expression of the reporter gene carrying the promoter of *MRC1* in a dose-dependent manner, as evidenced by increased MRC1-luciferase activity (Fig. 5G). As expected, in the presence of TNFα, MRC1-luciferase activities in chNrf2-overexpressing cells were significantly enhanced. To confirm Nrf2 activation during the development of plexiform lesions, lung tissues were subjected to immunohistochemistry analysis (Additional file 3: Fig. S2). The immunostaining intensity for Nrf2 in mature lesions was stronger than that in early lesions. In addition, in contrast to the fact that Nrf2 was localized predominantly in the cytoplasm of cells in the early lesions, Nrf2 was found to be located predominantly in the nucleus of the foam-like macrophages present in more mature ones (Additional file 4: Fig. S3).

### Intratracheal TNFα installation enhances the formation of plexiform lesions

**Fig. 6:**
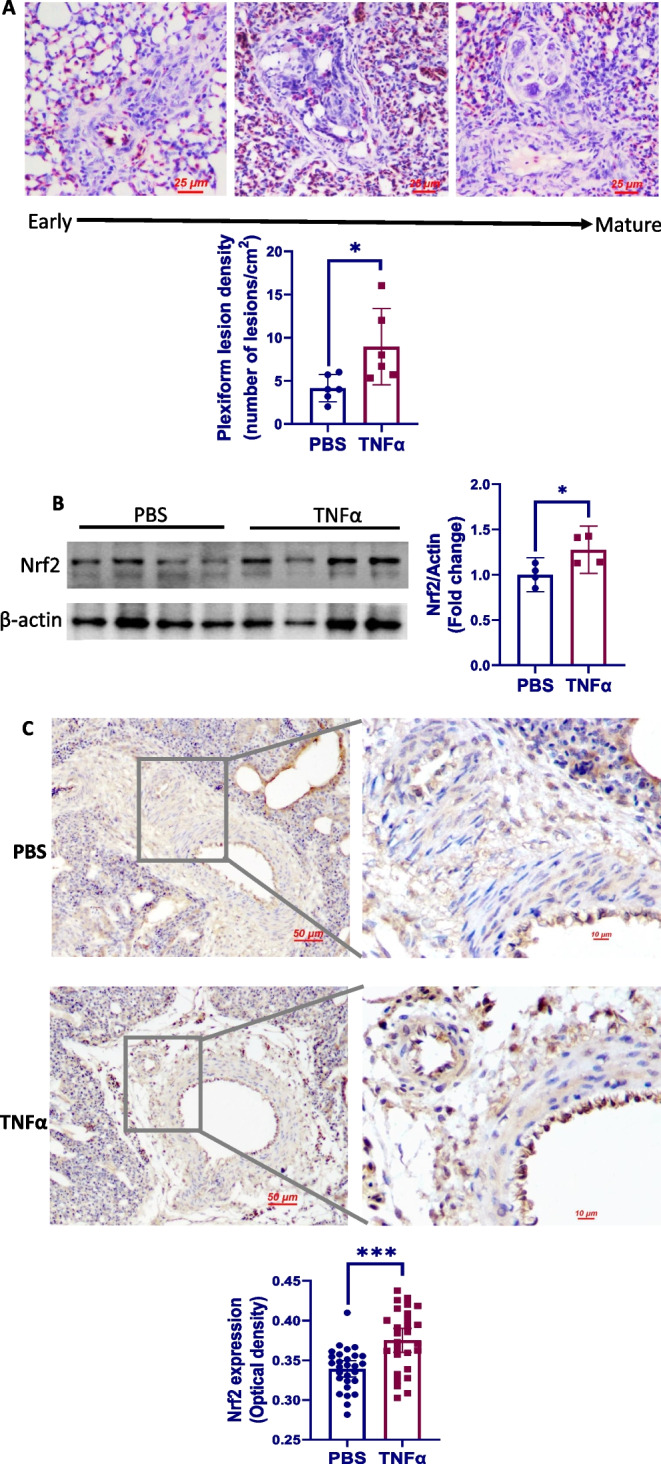
Intratracheal TNFα installation enhances the formation of plexiform lesions. **A** Representative photographs showing the morphology of plexiform lesions in the lung of TNFα-treated birds. Plexiform lesion density was expressed as the number of lesions per section/cm^2^ per section (*n* = 6). **B** Western blots and densitometry analyses of Nrf2. Total proteins extracted from lung tissue were analyzed by immunoblotting with anti-Nrf2 antibody. β-actin are shown as loading control. Densitometry data represent the mean ± 95% confidence interval of 4 birds and are representative of 2 separate experiments. **C** Immunohistochemistry staining of Nrf2 in lung tissue. Expression of endothelial Nrf2 was semi-quantified by measuring the optical density (OD) in 28 pulmonary arterioles randomly selected from 6 birds in each group

As expected, local TNFα administration resulted in a significant increase in the number of plexiform lesions that were morphologically undistinguishable from those observed in the control group (Fig. 6A). Western blot analysis demonstrated increased amount of Nrf2 protein in the lung of TNFα-treated birds as compared to the controls, suggesting an overactivation of Nrf2 in response to TNFα (Fig. 6B). Consistent with the Western blot analysis data, birds treated with TNFα had increased endothelial Nrf2 expression as assessed by immunohistochemistry staining (Fig. 6C, D). TNFα administration also led to an elevation in RV/TV ratio, although the difference between groups was not statistically significant (Fig. 6E).

## Discussion

In the present study, we described several findings supporting a view that eEPCs undergo macrophage differentiation during the development and evolution of plexiform lesions in our avian model. First, we have demonstrated that the cells in the early lesions have a typical eEPC phenotype (i.e., a mixed macrophage/endothelial cell phenotype) whereas those predominated in more mature lesions display molecular and morphological characteristics of macrophages. In addition, we show that chronic inflammation induces the differentiation of eEPC into macrophage lineage, resulting in reduced angiogenic potential. Furthermore, we provide direct evidence that local administration of TNFα produces plexiform lesions. Our findings are important, regarding that the morphological and molecular characteristics and anatomic distributions of plexiform lesions developed in broilers closely resemble that seen in human PAH patients [34, 38].

PAH is a common cardiovascular disorder in modern broiler chickens, with an estimated incidence of 3% in all broilers reared under normal conditions that promote rapid growth. Although the primary triggers of PAH in broilers remain unclear, accumulating evidence suggests that anatomically insufficient lung volume predisposes broilers to PAH [54, 55]. Unlike that in mammals, avian pulmonary capillaries are rigid tubes that prevent them from expanding in response to increased blood flow. The result is that pulmonary arterial pressure rises linearly with pulmonary blood flow [56]. Rapid growth in broilers incurs progressive increases in cardiac output that inevitably lead to increased pulmonary arterial pressure due to the corresponding increases in pulmonary blood flow. Research has shown that pulmonary arterial pressure in normal broilers increases from 20 to 25 mmHg between 2 and 3 weeks and maintains at approximately 25 mmHg during weeks 4 and 5 of age [57].Individuals having the most restricted pulmonary vascular capacity develop PAH when their right heart must develop an excessively elevated pulmonary arterial pressure to overcome the increased resistance to flow through the constricted pulmonary arterioles [55]. Sustained PAH triggers a series of events leading to structural vascular changes similar to the observations in human patients, including medial thickening and intimal proliferation [43, 58, 59]. Despite that fact that PAH broilers demonstrate increased plexiform lesions in their lungs [60], we and others have shown that PAH is not an essential prerequisite of the structures [38, 39, 42]. Nevertheless, an elevation in pulmonary arterial pressure appears to play a role in the process [41]. It is now believed that increases in pulmonary arterial pressure may create turbulent flow in increasing numbers of branch points or curved portions, leading to increased plexiform lesions as observed in PAH broilers [60]. The importance of disturbed flow for the development of plexogenic pulmonary arteriopathy is indirectly supported by the fact that the lesions can hardly be recapitulated in rodents in which turbulent flow rarely occurs because of the rapid stabilization of flow in small vessels [61].

eEPCs have been recognized as monocyte-derived circulating angiogenic cells [18] and are known to home to the sites of vascular injury for endothelial repair [62]. We have previously reported the accumulation of eEPCs (CD133+ and VEGFR-2+ cells) in the early lesions located at branch points of interparabronchial arterioles in broiler lungs [42]. In the present study, we extended our previous findings by demonstrating the expression of a macrophagic marker MRC1 in these cells, further confirming their eEPC phenotype [63]. This finding is important, regarding the presence of fully differentiated macrophages in the mature lesions.

In compliance with the hypothesis that chronic inflammation contributes to the pathology of plexiform lesions, we determined increased endothelial TNFα expression in the parent arterioles of plexiform lesions and constant mononuclear cell infiltrates around the lesions. We next used an in vitro model to test our hypothesis that chronic inflammation induces macrophage differentiation of eEPC resulting in reduced angiogenic potential. In line with an early study where incubation with TNFα showed no effect on eEPC death [64], we did not observe a significant effect of TNFα at 10–100 ng on cell viability. As expected, prolonged TNFα stimulation promoted the differentiation of eEPCs to macrophages, as evidenced by decreased expression in CD133 and increased expression in MRC1 together with the acquisition of strong phagocytotic activity. Additional experiments were carried out to examine if chronic inflammation leads to a conversion of eEPCs to macrophages in vivo. For this, eEPCs were mixed with Matrigel containing TNFα and were subcutaneously injected into broiler chickens to allow neovascularisation to develop for 6 days. Results from these studies demonstrate that eEPCs undergo macrophage differentiation in Matrigel plugs containing TNFα, resulting in reduced neovascularization in Matrigel plugs. Together, the data provide clear evidence that chronic inflammation predisposes eEPCs to differentiate into macrophage lineage, a process that impairs the angiogenic potential of eEPCs. In context, our results allow us to argue that the development of plexiform lesions is attributed to the impairment of angiogenic processes that is associated with the switching of eEPCs to macrophages in response to chronic inflammation.

To explore the potential mechanisms by which TNFα promotes eEPC-to-macrophage conversion, we focused on Nrf2, a well-known oxidative stress-responsive transcription factor. Interestingly, TNFα exposure had no significant effect on Nrf2 activation in eEPCs during the first 3 days, suggesting an intrinsic capability of these cells to tolerate inflammatory stimulation [65]. However, Nrf2 activation was evident at day 6 following TNFα exposure, a time point at which increased MRC1+ macrophages were present in the cultures. We found that Nrf2 hyperactivation was sufficient to induce the differentiation of eEPC to macrophage lineage. In support of our findings, Nrf2 has also been shown to regulate the differentiation of other cell types [66, 67]. In addition, we found that Nrf2 was able to bind to the promoter of *MRC1* to trigger its expression. By using a luciferase assay, we confirmed that Nrf2 regulates TNFα-induced eEPC-to-macrophage conversion. Showing a very good agreement with the in vitro data, Nrf2 protein was found to be barely expressed in the cells within the early plexiform lesions, whereas its expression was strong in more mature ones. Taken together, it is reasonable to suggest that local Nrf2 hyperactivation contributes to the pathogenesis of plexiform lesions by inducing macrophage differentiation of eEPCs. Further studies are warranted to test whether targeting Nrf2 would be beneficial for intervention of the lesions.

The present study has several limitations. First, since specific surface markers for the identification of macrophage subpopulations in avian species have not been defined, subgroups of the macrophages in the plexiform lesions and in the in vitro cultures challenged with TNFα were not specified. Second, the present study focused only on the eEPCs in the pathogenies of plexiform lesions. We acknowledge that TNFα may also stimulate endothelial-to-mesenchymal transition to promote the process [68]. Third, we did not evaluate the effect of TNFα installation on pulmonary blood flow and pulmonary vascular resistance. Thus, we could not conclude that the moderate increase in pulmonary arterial pressure as measured by RV/TV ratio after TNFα administration is merely related to the increased formation of plexiform lesions.

## Conclusions

In summary, the current study provides evidence supporting the view that local inflammatory cytokine TNFα plays a critical role in the development of plexiform lesions. Our observation that chronic exposure to TNFα promotes the phenotypic switching of eEPCs towards macrophage lineage might explain why the eEPCs loss their angiogenic activities after homing to the sites of vascular injury within the pulmonary vasculature and the origin of macrophages in the lesions. We show for the first time that Nrf2 drives the phenotypic switching of eEPCs towards macrophages following chronic TNFα stimulation. Taken together, understanding of these mechanisms that drive the development of plexiform lesions may pave the way to provide novel therapeutic strategies for the treatment of PAH patients.

## Supplementary Information

**Additional file 1: Table S1.** Primers information.

**Additional file 2: Fig. S1.** Effect of TNFα on eEPC viability.

**Additional file 3: Fig. S2.** Nrf2 activation during the development of plexiform lesions.

**Additional file 4: Fig. S3.** The effect of TNFα administration on RV/TV ratio of broilers.

## Data Availability

The datasets used and/or analysed during the current study are available from the corresponding author on reasonable request.

## Abbreviations

CAC: Circulating angiogenic cells
Dil-Ac-LDL: 1,1-Dioctadecyl-3,3,3,3-tetramethylindocarbocyanine-labelled acetylated low-density lipoprotein
eEPCs: Early progenitor endothelial cells
H&E: Haematoxylin and eosin
Keap1: Kelch-like ECH-associated protein 1
Lectin: Ulex europaeus agglutinin
Nqo-1: NAD(P)H:quinone oxidoreductase 1
Nrf2: Nuclear factor erythroid 2-related factor 2
PAH: Pulmonary arterial hypertension
PBMC: Peripheral blood mononuclear cell
TNFα: Tumor necrotic factor-α
VEGFR-2: Vascular endothelial growth factor receptor-2
