# Supplementary material for: Potential contribution of early endothelial progenitor cell (eEPC)-to-macrophage switching in the development of pulmonary plexogenic lesion
Source: Respir Res. 2022 Oct 23;23:290. doi: 10.1186/s12931-022-02210-7 (PMC9590182; doi:10.1186/s12931-022-02210-7)
Supplement: Supplementary file 1 — Additional file 1: Table S1. Primers information. [file 12931_2022_2210_MOESM1_ESM.docx]

| Gene products | Primer sequence 5’→3’  (forward/reverse) | mRNA accession No. | Amplicon size (bp) |
| --- | --- | --- | --- |
| B2M | TCCTTCAACGACGACTGGAC | NM_001001750.4 | 146 |
|  | GGCACAGCTCAGAACTCGG |  |  |
| RPL19 | AGACCAACGAGATCGCCAAC | NM_001030929.1 | 126 |
|  | GGGCCAAGGTGTTCTTCCTG |  |  |
| KEAP1 | ACTTCGCTGAGGTCTCCAAG | XM_025145847.1 | 142 |
|  | CAGTCGTACTGCACCCAGTT |  |  |
| NQO1 | ACCTCTTTCAACCACGCCAT | NM_001277620.2 | 139 |
|  | TCTTGAGGGGTCCGGTGAT |  |  |
| MRC1 | AAGCACTCTCTGGTGTGCAA | NM_001319013.2 | 89 |
|  | AGTCTTTGTGGAGAGTGTCTTT |  |  |
| CD133 | CGAACCTGGGCCAATAGGAA | XM_004936155.2 | 145 |
|  | GATGGCCTTCTGATAATCGCC |  |  |

**Table S1. Primer sets used in SYBR-green-based quantitative real-time PCR (qPCR)**
