# Supplementary material for: Potential contribution of early endothelial progenitor cell (eEPC)-to-macrophage switching in the development of pulmonary plexogenic lesion
Source: Respir Res. 2022 Oct 23;23:290. doi: 10.1186/s12931-022-02210-7 (PMC9590182; doi:10.1186/s12931-022-02210-7)
Supplement: Supplementary file 2 — Additional file 2: Fig. S1. Effect of TNFα on eEPC viability. [file 12931_2022_2210_MOESM2_ESM.pdf]

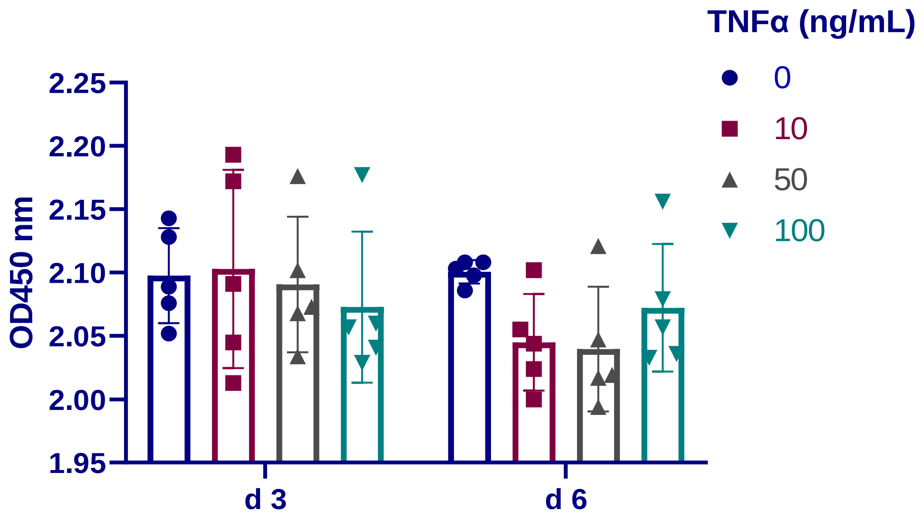

Figure S1. Effect of TNFα on eEPC viability.

Early EPCs (eEPCs) were seeded in 96-well plates at 1× 10^4 per well and incubated with TNFα at 0–100 ng/mL for the indicated time. Cell viability was determined by using Cell Counting Kit-8 (CCK-8) (n = 5). Data were expressed as mean ± s.d. (ANOVA).
