# Supplementary material for: Potential contribution of early endothelial progenitor cell (eEPC)-to-macrophage switching in the development of pulmonary plexogenic lesion
Source: Respir Res. 2022 Oct 23;23:290. doi: 10.1186/s12931-022-02210-7 (PMC9590182; doi:10.1186/s12931-022-02210-7)
Supplement: Supplementary file 3 — Additional file 3: Fig. S2. Nrf2 activation during the development of plexiform lesions. [file 12931_2022_2210_MOESM3_ESM.pdf]

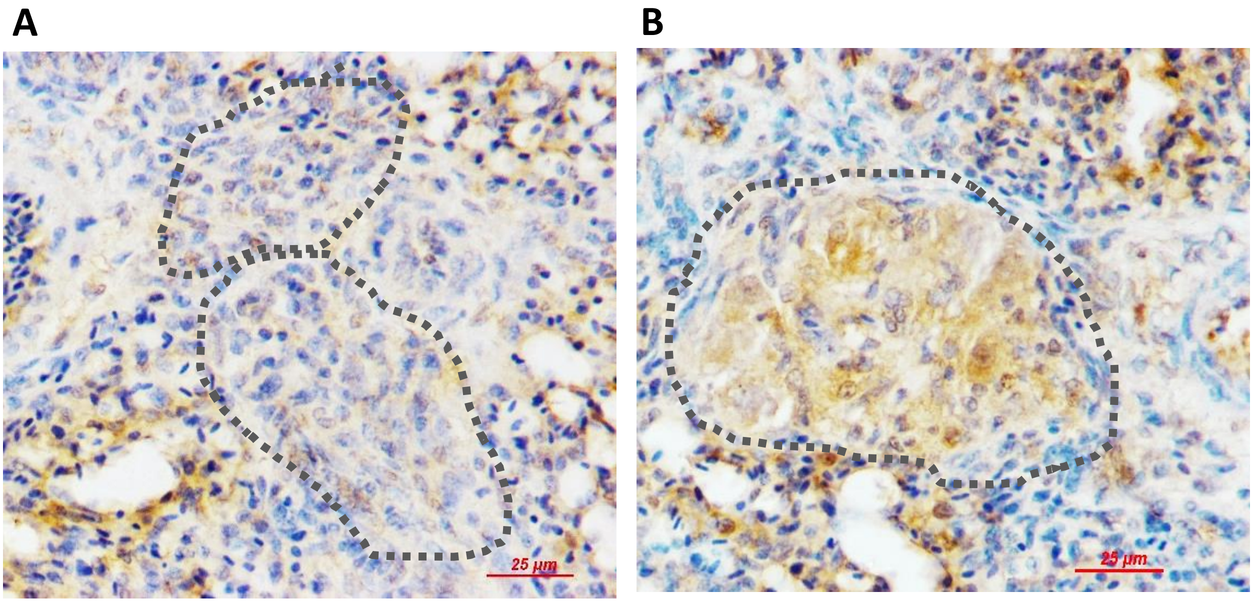

Figure S2. Nrf2 activation during the development of plexiform lesions

Representative immunohistochemistry photographs showing the expression of Nrf2 in immature (A) and a more mature lesion (B). Immunohistochemistry analysis was performed using a primary antibody against Nrf2. All immunostained sections are counterstained with hematoxylin. Note that Nrf2 protein was localized predominantly in the cytoplasm of cells in the early lesion (A) whereas distributed in both the cytoplasm and nucleus in the foam-like macrophages in more mature lesion (B). The dotted lines represent the edge of lesions.
