# Supplementary material for: Potential contribution of early endothelial progenitor cell (eEPC)-to-macrophage switching in the development of pulmonary plexogenic lesion
Source: Respir Res. 2022 Oct 23;23:290. doi: 10.1186/s12931-022-02210-7 (PMC9590182; doi:10.1186/s12931-022-02210-7)
Supplement: Supplementary file 4 — Additional file 4: Fig. S3. The effect of TNFα administration on RV/TV ratio of broilers. [file 12931_2022_2210_MOESM4_ESM.pdf]

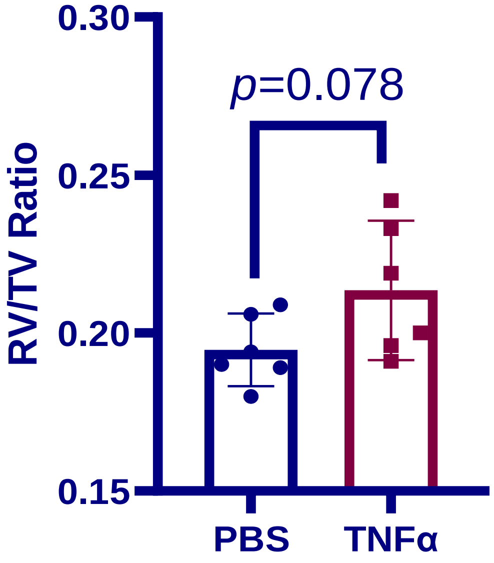

Figure S3. The effect of TNFα administration on RV/TV ratio of broilers.

Broiler were instilled intratracheally with 0.5 µg TNFα for two doses with an interval of 3 d. Lung samples were collected 3 d after the second dose. The hearts were weighed for calculation of the right-to-total ventricular weight ratio (RV/TV) as an indicator of pulmonary arterial hypertension (PAH). Data are expressed as mean ± 95% confidence interval (*n* = 6).
